# Increase of Productivity and Neutralization of Pathological Processes in Plants of Grain and Fruit Crops with the Help of Aqueous Solutions Activated by Plasma of High-Frequency Glow Discharge

**DOI:** 10.3390/plants10102161

**Published:** 2021-10-12

**Authors:** Yuri K. Danilejko, Sergey V. Belov, Alexey B. Egorov, Vladimir I. Lukanin, Vladimir A. Sidorov, Lyubov M. Apasheva, Vladimir Y. Dushkov, Mikhail I. Budnik, Alexander M. Belyakov, Konstantin N. Kulik, Shamil Validov, Denis V. Yanykin, Maxim E. Astashev, Ruslan M. Sarimov, Valery P. Kalinichenko, Alexey P. Glinushkin, Sergey V. Gudkov

**Affiliations:** 1 Prokhorov General Physics Institute of the Russian Academy of Sciences, 119991 Moscow, Russia; dyuk42@list.ru (Y.K.D.); ser79841825@yandex.ru (S.V.B.); a261@rambler.ru (A.B.E.); vladimirlukanin@yandex.ru (V.I.L.); 2017vovas@gmail.com (V.A.S.); ya-d-ozh@rambler.ru (D.V.Y.); astashev.max@gmail.com (M.E.A.); rusa@kapella.gpi.ru (R.M.S.); 2 Semenov Institute of Chemical Physics of Russian Academy of Sciences, 119991 Moscow, Russia; apasheva@rambler.ru (L.M.A.); vdushkov@yandex.ru (V.Y.D.); ziraf@mail.ru (M.I.B.); 3 Federal Scientific Center for Agroecology, Integrated Land Reclamation and Protective Afforestation of the Russian Academy of Sciences, 400062 Volgograd, Russia; dokbam49@mail.ru (A.M.B.); kulikn@yandex.ru (K.N.K.); 4 Federal Research Center Kazan Scientific Center of Russian Academy of Sciences, 420008 Kazan, Russia; sh.validov@knc.ru; 5 All-Russian Phytopathology Research Institute, 143050 Big Vyazyomy, Russia; kalinitch@mail.ru (V.P.K.); glinale@mail.ru (A.P.G.)

**Keywords:** high-frequency glow discharge, low temperature plasma, fusarium, PAW, germination of seeds, plant resistance

## Abstract

In this work, we, for the first time, manufactured a plasma-chemical reactor operating at a frequency of 0.11 MHz. The reactor allows for the activation of large volumes of liquids in a short time. The physicochemical properties of activated liquids (concentration of hydrogen peroxide, nitrate anions, redox potential, electrical conductivity, pH, concentration of dissolved gases) are characterized in detail. Antifungal activity of aqueous solutions activated by a glow discharge has been investigated. It was shown that aqueous solutions activated by a glow discharge significantly reduce the degree of presence of phytopathogens and their effect on the germination of such seeds. Seeds of cereals (sorghum and barley) and fruit (strawberries) crops were studied. The greatest positive effect was found in the treatment of sorghum seeds. Moreover, laboratory tests have shown a significant increase in sorghum drought tolerance. The effectiveness of the use of glow-discharge-activated aqueous solutions was shown during a field experiment, which was set up in the saline semi-desert of the Northern Caspian region. Thus, the technology developed by us makes it possible to carry out the activation of aqueous solutions on an industrial scale. Water activated by a glow discharge exhibits antifungicidal activity and significantly accelerates the development of the grain and fruit crops we studied. In the case of sorghum culture, glow-discharge-activated water significantly increases drought resistance.

## 1. Introduction

Increasing the yield and improving the quality of agricultural products, reducing the costs of its production is impossible without the introduction of economically profitable and environmentally friendly technologies that ensure an increase in the productivity of agricultural plants. One of the promising directions in the creation of such technologies is the use of biologically activated water [1,2,3,4,5,6,7]. Usually, to obtain activated aqueous solutions, devices are used, the principle of operation of which is based on electrochemical methods using electrolyzers of various designs, including the separation of water into the constituents “anolyte” and “catholyte” [7,8]. In recent years, a method for activating water, as well as an aqueous solution of various electrolytes, has become widespread by exposing its surface to a low-temperature non-equilibrium plasma generated by a high-voltage electric discharge in an atmosphere of various gases [9,10], a microwave discharge in an argon atmosphere [11,12], high-voltage discharge in the volume of water under conditions of intense cavitation [13,14].

An alternative approach is the activation of solutions using a high-frequency glow discharge in water vapor [15,16,17]. Glow discharge is a type of stationary self-sustaining electric discharge, which is formed at low pressure and low current. Generally, a glow discharge is produced in gases at low pressure. The main characteristics of a glow discharge remain relatively stable over time [18]. In a glow discharge, the gas conducts electricity well due to its strong ionization. Gas ionization is achieved through the emission of electrons from the cathode under the action of a strong electric field (or high temperature). In addition, the secondary electron emission of electrons from the cathode, caused by the bombardment of the cathode with positively charged ions of the medium. In general, a glow discharge can be maintained at a voltage significantly lower than the dielectric breakdown voltage of the medium [18]. In recent years, methods have been developed for obtaining a glow discharge in liquids. In this case, the decomposition of water vapor occurs due to the transfer of energy from an electron gas with a temperature not higher than 5 eV to water molecules. Water decomposition can proceed through two channels: a channel through stepwise excitation of vibrational degrees of freedom of water molecules and a channel through the dissociative attachment of electrons to water molecules [19]. In both cases, they form reactive oxygen and nitrogen species with significant biological activity. Activation of water using a glow discharge is the most economically viable approach, this approach is easily scalable and allows you to handle large volumes of liquids [20].

The main goal of this work was to create a plasma-chemical reactor operating at frequencies of several tens of kilohertz. We assumed that such a reactor could process aqueous solutions more efficiently, which ultimately would allow obtaining activated water at a lower cost. We also assume that in a high-frequency plasma-chemical reactor, we can obtain equilibrium peroxide concentrations of more than 1 mM. Solutions with similar concentrations of biologically active substances can be used to combat phytopathogens. In addition, one of the objectives of the study was the use of the obtained activated aqueous solutions in agricultural practice.

In this work, we, for the first time, manufactured a plasma-chemical reactor operating at a frequency of 0.11 MHz. The reactor allows for the activation of large volumes of liquids in a short time. The physicochemical properties of activated liquids (concentration of hydrogen peroxide, nitrate anions, redox potential, electrical conductivity, pH, concentration of dissolved gases) are characterized in detail. Antifungal activity of aqueous solutions activated by a glow discharge was investigated. It was shown that aqueous solutions activated by a glow discharge significantly reduced the degree of presence of phytopathogens and their effect on germination of such seeds. Seeds of cereals (sorghum and barley) and fruit (strawberries) crops were studied. The greatest positive effect was found in the treatment of sorghum seeds. Moreover, laboratory tests showed a significant increase in sorghum drought tolerance. The effectiveness of the use of glow-discharge-activated aqueous solutions was shown during a field experiment, which was set up in the saline semi-desert of the Northern Caspian region. Thus, the technology developed by us makes it possible to carry out the activation of aqueous solutions on an industrial scale. Water activated by a glow discharge exhibits antifungicidal activity and significantly accelerates the development of the grain and fruit crops we studied. In the case of sorghum culture, glow-discharge-activated water significantly increases drought resistance.

## 2. Results

### 2.1. Physicochemical Properties of Aqueous Solutions Activated by Glow Discharge Plasma

The influence of the plasma of a glow discharge on the physicochemical parameters of water was investigated (Table 1). It was found that the change in the physicochemical parameters of water occurs within 40–50 min of treatment. With further processing, the rate of change of the parameters significantly decreases or, as in the case of hydrogen peroxide, it ceases to change, reaching a stationary value of 7 × 10^−3^ M.

During processing, the conductivity of the solution increased almost linearly. The rate of increase in the specific conductivity of the solution was approximately 450 μS/cm/min. In this case, the concentration of molecular oxygen dissolved in water tends to decrease. At the same time, the decrease in the concentration of molecular oxygen during the first 20 min of treatment was much more intense than during the next 20 min. In addition, during the activation process, the pH of the solution changes. During the first 20 min of treatment, the pH value increased by almost one. With further activation within 20 min, the pH value changed only by 0.5 units. Similar changes were observed in the values of the redox potential. In the first 20 min of processing, the potential increased by almost 200 mV. With subsequent processing, an increase of less than 100 mV was observed. During activation, nitrate anions accumulate in aqueous solutions. The increase in the concentration of nitrate anions linearly depends on the processing time. It should be noted that, in contrast to other studied parameters, the concentration of nitrate anions continues to increase after 40 min of exposure. Accumulation of nitrate anions was not observed when experiments were carried out in an argon atmosphere. The increase in the concentration of hydrogen peroxide was linearly dependent on the processing time. The above-described patterns were applicable at processing times of 40–50 min. With further activation of aqueous solutions, the concentration of hydrogen peroxide ceased to change significantly. In some experiments, a decrease in the concentration of hydrogen peroxide was observed. We carried out a number of experiments using electrolytes based on salts of phosphoric, nitric, and a number of organic acids. It was shown that the generation of hydrogen peroxide substantially depends on the composition and concentration of the starting compounds in an aqueous solution. In addition, depending on the composition and concentration of salts, the duration of the activity of the resulting solution depends. Basically, to assess the preservation of the activity of the plasma-activated solution, we used the concentration of hydrogen peroxide. It was shown that under different conditions, the concentration of hydrogen peroxide decreased by two times within 1–3 weeks. Based on the results obtained, in further studies, we used activated solutions containing the maximum amount of active substances (treatment time 40 min).

### 2.2. Fungicidal Properties of Aqueous Solutions Activated by Glow Discharge Plasma

The fungicidal properties of aqueous solutions activated by glow discharge plasma were studied using healthy (−) and fusarium-infected (+) seeds of *Sorghum bicolor* (L.) *Moench*, wheat *Triticum aestivum*, and strawberry *Fragaria* L. The proportion of affected seeds according to microscopy data was 98, 92, and 76% for wheat, sorghum, and strawberry, respectively. Fusarium blight was identified by the presence of the pathogens *Fusarium* sp. by microscopy (Table 2). It was shown that soaking seeds in deionized water did not significantly reduce the presence of fungus on the surface of the grains. Soaking the seeds for 5 h in the activated solution led to a significant decrease in the degree of seed infestation. In some cases, using microscopy, it was difficult to distinguish the degree of seed infestation, as well as to reliably identify the type of phytopathogen. Moreover, the microscopy we used did not allow us to distinguish between viable and non-viable forms of the fungus.

The infection was verified by RT-PCR. In samples of sorghum (+) and wheat (+) cereals, the DNA of *F. avenaceum* (*C*t ~10) and *F. graminearum* (*C*t ~17) was identified. In samples of all cereals (-), DNA of both species was not detected. *F. oxysporum* DNA (Ct ~29) was identified in strawberries by real-time PCR. Soaking seeds in deionized water did not significantly affect the concentration of pathogen DNA. After soaking in a solution activated by glow discharge plasma, the concentration of the original DNA of the pathogen significantly decreased. The best results have been achieved with sorghum seeds. Treatment of strawberry seeds was slightly less effective. The least effective treatment was the wheat seed. The presence or absence of pathogen DNA cannot answer the question of the viability of the pathogen. In this regard, seed germination was carried out. It was shown that among all the studied crops, the largest percentage of Fusarium-infected seeds of the treated activated solutions germinates in sorghum; strawberry treatment was slightly less effective. Wheat seed treatment yielded the least significant results.

The results obtained do not allow determining the reasons for the better germination and germination of seeds treated with activated water. On the one hand, of course, activated water has a fungicidal effect on fusarium. On the other hand, activated water can contribute to the development of increased resistance of crops to fungal and bacterial diseases. To answer this question, a series of experiments with healthy seeds was carried out. Since the treatment of sorghum seeds turned out to be the most effective, we carried out further tests only on sorghum seeds.

### 2.3. Study of the Effectiveness of the Action of Activated Water on the Yield of Sorghum Crops (Laboratory Tests)

The main goal of the experimental studies was to assess the effect of the activated solution at all stages of plant growth and development. To solve this problem, research was carried out in laboratory and field conditions. In the first treatment variant, sorghum seeds were soaked in the activated solution without dilution. To find the optimal processing time, sorghum seeds in the amount of 100 pcs. were soaked in the test solution for a certain time, then washed in distilled water and germinated at 25 °C in Petri dishes on filter paper moistened with distilled water. After 48 h from the start of the treatment, the number of hatching seeds was measured. The seed was considered to have hatched if the root length at the time of measurement was more than 5 mm. The experiment was carried out in triplicate. The average value of seed germination energy depending on the treatment time is presented in Table 3. It is shown that the optimal seed treatment time was in the range of 4–7 h, with the best indicator in the region of 5 h. Soaking seeds in a solution for more than 11 h leads to a decrease in germination. In the future, we would use sorghum seeds, pre-moistened for 5 h with activated water without dilution (the best option, Table 4). Germination of treated infected seeds in Petri dishes on filter paper moistened with distilled water in all replicates did not lead to mold formation, even on dead, not germinated seeds. In control experiments with distilled water, non-germinated seeds were covered with mold after 36 h.

In the second variant, laboratory tests were carried out in soil on a Klasmann peat mixture, recipe “883”. This soil was optimal for the cultivation of young greenery due to the low concentration of minerals and the presence of perlite in its composition. Sorghum seeds in the amount of 100 pcs. were soaked for 5 h in an activated solution without dilution, then washed in distilled water and sown in a container with soil with a volume of 0.7 L to a depth of 5 cm. After 120 h from the moment the seeds were soaked, the young sprouts were carefully washed from the soil, after which the average full germination of seeds, the average length of the main root, and the average weight of the sprout, taking into account the hypocotyl, were measured. The experiment was carried out in triplicate, and the averaged data are shown in Table 4.

When seeds were treated with water activated by glow discharge plasma, seedlings appeared 12 h earlier than control. Such a temporary reserve allowed plants to start the process of photosynthesis earlier, which means that they quickly begin to build up the root system and vegetative mass. Thus, the length of the root in the group of plants treated with activated water was 20% longer than in the control. The biomass of one plant in the group of plants treated with activated water was 25% higher than in the control group. It should be noted that in the group of plants treated with activated water, the standard errors of the mean were less than in the control group.

**Figure 1:**
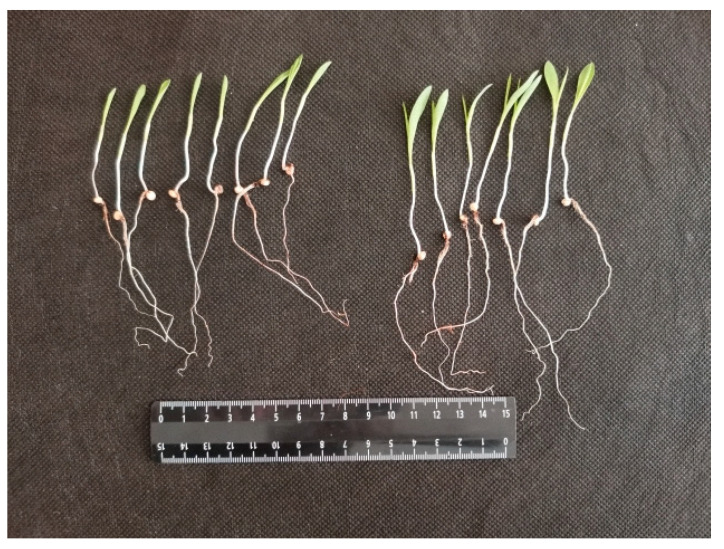
Photographs of randomly selected young shoots of sorghum on the 5th day after seed treatment (**left**—control, **right**—experiment (seeds were soaked in water with activated glow discharge plasma for 5 h)).

Figure 1 shows examples of sorghum sprouts on the 5th day from the moment of seed treatment in control and experiment. In the experiment, a more developed leaf surface of young shoots was clearly observed. If we express the leaf surface area in numbers, then in the group of plants treated with activated water, the leaf area will be 2.5 times larger than in the control.

**Figure 2:**
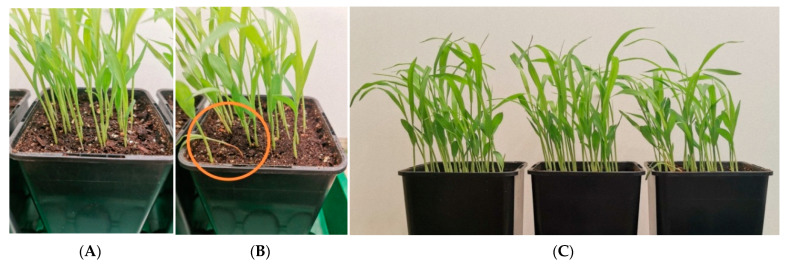
General view of sorghum plants on the 10th day after seed treatment (**A**,**C**) (pot in the center)—treated with activated water, (**B**,**C**) (left and right pots)—control. An area of infection and spread of root rot is highlighted around.

Figure 2 shows the results of an experiment in soil under conditions of provoking root rot (thickened sowing—40 seeds per pot with a volume of 0.7 L), excessive soil moisture and watering with cold water, low air temperature. Sorghum seeds were pre-moistened for 5 h in activated water. Distilled water was used as a control. After 10 days from the moment of seed treatment, root rot was recorded only in the control samples. The source of phytopathogens, apparently, are the seeds themselves, and this factor, among other things, negatively affects the varietal and sowing qualities of seeds.

In the third variant, the moistening of seeds with an activated solution was carried out with different dilutions with deionized water and an increase in the time of seed treatment. In this experiment, 100 sorghum seeds (in 3 replicates) were laid out on filter paper moistened with the test solution with a supposed promising dilution and germinated in Petri dishes at a temperature of 25 °C. After 10 days, the main parameters of the seedlings were measured. Seed energy was assessed by the length of the main root and germination (Table 5).

**Figure 3:**
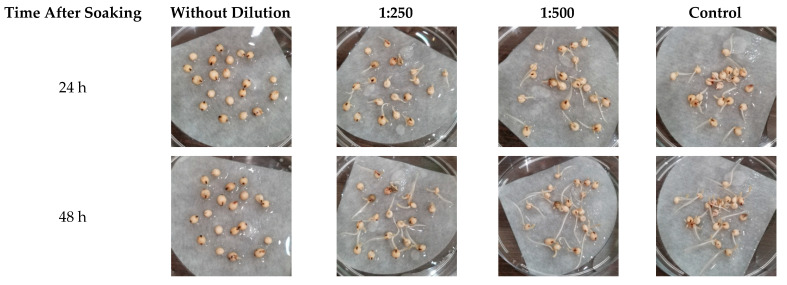
Photographs of sorghum seedlings at different dilutions of activated water and soaking times.

As can be seen from Table 5, the optimal dilution ratio of the activated solution for sorghum seeds when soaked for 1 or 2 days was 1:500. With this dilution, even on the first day of germination, a more developed main root and higher germination energy were visually recorded. This effect was most pronounced 24 h after seed treatment (Figure 3).

Thus, the optimal dilution ratio of the activated solution for sorghum seeds for long periods of soaking corresponds to 1:500. When diluted optimally, the activated water stimulates the development of sorghum and to some extent, increases the vitality of the plants. The high concentration of activated water and the long exposure time inhibit the growth of sorghum. With a long exposure time, the activated solution acts as an antiseptic.

The variant with the use of activated water without dilution may be promising for disinfection and protection of plants from diseases. In the selection and seed production of agricultural crops, or in areas related to microclonal reproduction of plants. The variant with the dilution of activated water is preferable in the conditions of industrial cultivation of crops. This option significantly reduces the need for activated water, eliminating the effect of inhibition.

### 2.4. Study of the Effectiveness of the Action of Activated Water on the Yield of Sorghum Crops (Field Trials)

The effect of the pre-sowing treatment of seeds with activated water was observed during the entire cycle of plant growth. At the first stage, seed germination was monitored. After sowing, performed on 19 May, from 20.05 to 26.05 precipitation with an intensity of 1–2 mm fell several times. In the control variant, the crops emerged on the 6th day (standard 7–8 days). The seeds, treated with activated water, sprouted more amicably a day earlier.

Taking into account the specificity of sorghum growth, the slow development of the aerial part in the first weeks with enhanced development of the root system, observations of the second stage were carried out 20 days after sowing (08.06), during the formation of the 5th leaf. Measurements of the height of the aboveground part recorded a statistically significant increase in the growth of sorghum in the activation variant by about 25%, which indirectly indicated a more intensive development of the root system.

**Figure 4:**
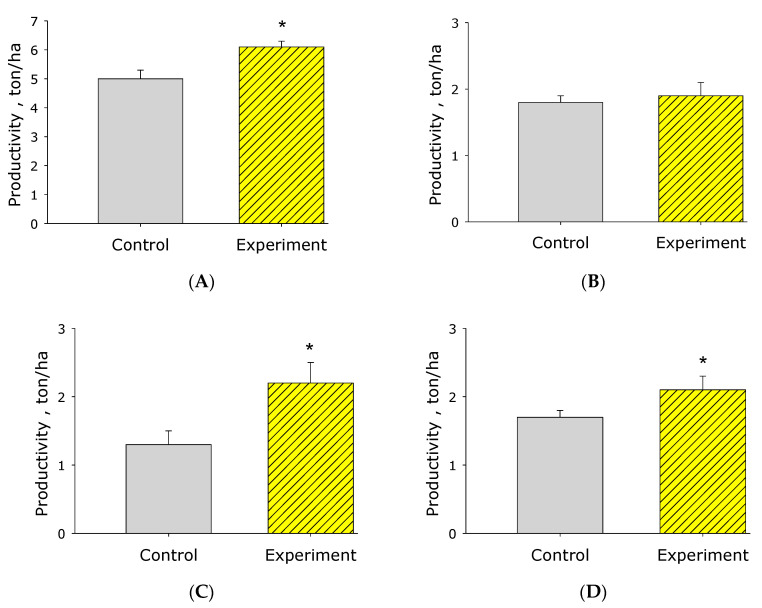
Histogram of the main results of the analysis of sheaves of biomass of grain sorghum “Eltonskoye” soaked in activated water (Experiment) and control water (Control). (**A**)—terrestrial biomass, ton/hectare; (**B**)—biomass of stubble 15 cm high, ton/hectare; (**C**)—hay biomass, ton/hectare; (**D**)—biomass of grain, ton/hectare. *—statistical differences relative to control (*p* < 0.05).

The third stage of observations was carried out at the end of the growing season (16.08) before harvesting with a combine using the standard sheaf selection method. According to the results of processing sheaves, a positive effect of pre-sowing seed treatment on the growth of biomass and grain yield was also recorded (Figure 4). As can be seen from the graph, grain yield increased according to biological accounting of sheaves by 10.6%; bunker accounting after harvesting with a combine by 10.4%. The mass of the aboveground part of sorghum plants (air-dry weight) upon activation increased by 19.6%, from 5.04 t/ha to 6.03 t/ha. The most effective treatment of seeds influenced the yield of hay, higher by 58.3% compared to the control. We explain this circumstance by the fact that the plants developed a deeper and more powerful root system, which intensified the growth of the shoots after a late rain shower (after 2 months of drought; at the end of July, about 50 mm fell). Intensive precipitation and the specificity of sorghum to give a fit had a very good effect on the yield of hay, control option—1.37 t/ha, version with activated water—2.19 t/ha. These indicators were quite high compared to the average.

## 3. Discussion

**Figure 5:**
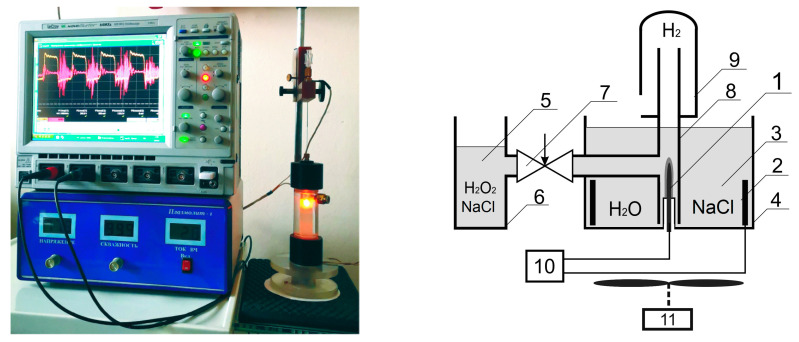
Photographs and schematic diagram of a reactor for obtaining an activated solution. Active electrode—1, neutral electrode—2, electrolyte solution—3, container for electrolyte—4, activated solution—5, container for activated Scheme—6. adjustable leak—7, quartz tube for overflow of activated solution—8, container for collecting hydrogen—9, high-frequency generator—10, magnetic stirrer—11.

**Figure 6:**
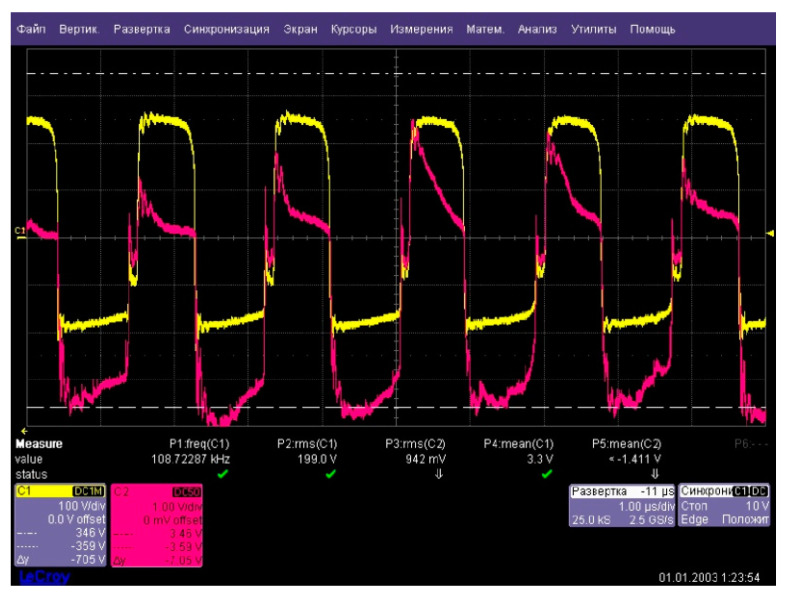
Oscillogram of current (red curve) and voltage (yellow curve) at the reactor input.

We have created a setup for the activation of aqueous solutions by a glow discharge plasma (Figure 5 and Figure 6). During the operation of the installation, a significant change in the physicochemical properties of aqueous solutions is observed (Table 1). With prolonged processing of liquids, the rate of change in the parameters of the solution is significantly slowed down. This is probably due to the following circumstances. As a result of the interaction of plasma with water vapor in water, atomic hydrogen and a hydroxyl radical are formed [21]. As a result of the recombination of these products in a liquid, molecular hydrogen (H^•^ + ^•^H→H_2_), hydrogen peroxide (OH^•^ + ^•^HO→H_2_O_2_), and water (OH^•^ + ^•^H→H_2_O) are formed [22]. With the accumulation of hydrogen peroxide in the solution, the reaction of its decomposition begins to play a significant role, which leads to the establishment of a stationary concentration of H_2_O_2_. In some cases, the equilibrium can be mixed, the process of decomposition of the formed hydrogen peroxide will be observed. Obviously, this process depends both on the type of the selected salt and on the storage method of the prepared solution [23].

In the process of activation of an aqueous solution by low-temperature plasma, hydrogen peroxide and nitrate anion are generated at the plasma-solution interface [24,25]. These compounds have significant biological activity, impart antiseptic, and disinfecting properties to an aqueous solution [26], which is shown in the manuscript (Table 1). It should be noted that hydrogen peroxide plays an important role in a number of processes important for plants [27]. In particular, hydrogen peroxide modulates the process of photosynthesis, affects the synthesis of starch and chlorophyll. If we go to the level of the whole plant, then hydrogen peroxide has a significant effect on the growth and development of plants [28,29,30,31]. It is known that pre-sowing treatment of seeds and foliar treatment of green plants with solutions of hydrogen peroxide in certain concentrations increase the stability and safety of agricultural plants in severe conditions of moisture deficit, frost, and soil salinity [32,33,34]. For this reason, plasma-activated aqueous solutions are effective and environmentally friendly stimulators of plant growth and vitality (Table 2, Table 3, Table 4 and Table 5, Figure 1, Figure 2, Figure 3 and Figure 4). After treatment with activated water, seeds have a greater energy potential, structural and functional rearrangements of membrane formations and macromolecules occur in them, as a result of which various biological changes occur in plants (Table 4 and Table 5). One of the main effects of biochemical exposure to an activated aqueous solution and other stressful influences is the generation of free radicals in plant seeds, which affect the enzymatic properties of seeds [35]. In addition, it was previously noted that this leads to an increase in the resistance of agricultural crops to fungal and bacterial diseases [36]. In addition, plants treated with activated water become more resistant not only to phytopathogens but also to drought [37]. It is the positive effect of activated water on the growth and resistance (survival) of plants in arid conditions that determined the feasibility of setting up a production experiment in extreme semi-desert conditions (Figure 4). We assume that the success of the experiment is related, among other things, to the peculiarities of the growth and development of the plant root system. It is known that hydrogen peroxide significantly affects the growth and development of the root system. This is due to the modification of the signal of the plant hormone auxin and changes in the expression of genes of the cell cycle [38]. The expediency of the experiment was also dictated by the well-known weak efficiency of fertilizers and growth stimulants in arid conditions due to a lack of moisture [39]. Previously, several laboratory studies were carried out on the effect of plasma-activated aqueous solutions on the drought resistance of plants. It was found that under conditions of water shortage when treating with plasma-activated water, the germination of *Vigna mungo* L. seeds increased by 10–15% [40]. Andreev et al. showed that plasma-activated water in laboratory conditions could improve the drought tolerance of seeds (10–20%) of radish [41], wheat [42], and other crops, as well as increase the rate of seed germination under drought conditions. A number of other laboratory examples are provided in the review article [43]. The activated solution obtained by the described method can be used in various sectors of the national economy, where activated water is traditionally used in agriculture for the treatment of seeds and adult plants, for foliar treatment of plants by spraying, as an antibacterial agent in animal husbandry, the food industry, etc.

## 4. Materials and Methods

### 4.1. Activated Water Generation Method

To activate the water, a reactor was used in which 2 activation methods were implemented, plasma-chemical and electrochemical. The schematic diagram of the reactor is shown in Figure 5. The design of the reactor was based on an electrochemical cell, including a vessel with electrolyte, in the volume of which 2 electrodes were immersed—active and passive. The active electrode was made of platinum and had the shape of a cylinder with a diameter of 0.5 mm and a length of up to 20 mm. The passive electrode was made of pyrolytic graphite and had an area of 8 cm^2^. The electrodes were connected to a specially designed high-frequency generator operating at 110 kHz. The working voltage on the electrodes was maintained in the range 250–350 V. In this case, the peak power consumed from the generator could reach 1500 W for a short time, which was enough to form a vapor-gas bubble on the active electrode and ignite a glow discharge in the vapor phase. The power of the device in a stationary mode was estimated depending on the composition of the aqueous solution at 100–300 W.

The oscillogram of the current flowing through the reactor is shown in Figure 2. It can be seen from the oscillogram that the current in the negative half-cycle (cathodic polarity) noticeably exceeded the current in the positive half-cycle (anodic polarity), while their shape was significantly different. This leads to the fact that the current flowing through the reactor had a constant component.

Thus, in the used reactor, 2 regions can be distinguished. The first region was formed by the gas phase, in which a high-frequency glow discharge burns in water vapor and plasma-chemical reactions take place. The electrodes for the plasma-chemical part of the reactor were the metal active electrode (cathode) and the plasma-electrolyte boundary (electrolyte anode). The second area was the electrolyte part, with a pulsating current with a constant component flowing through it, the electrodes of which were the plasma—electrolyte (electrolyte cathode) and passive (graphite) anode boundary. The presence of a constant current component leads to the appearance of electrochemical processes occurring in the electrolyte part of the reactor. As an electrolyte, a solution of sodium chloride with a concentration of 0.14 M was used. The volume of the experimental reactor was 200 cm^3^. The electrolysis of the solution proceeded without using a diaphragm. During electrolysis, the solution was intensively stirred using a magnetic stirrer. Upon reaching the operating mode, the temperature of the solution in the reactor stabilized at the level of 45 ± 2 °C.

We estimate the market value of the installation (generator, reactor) at $4200. The production costs of 1 L of activated water (electricity, distilled water, consumables) were $3–$5 (depending on the country, for example, in the Russian Federation, the price of one kilowatt of electricity is on average $0.05). Dilution of one liter of activated water makes it possible to obtain 500 L of working solution for seed dressing using the moistening method.

### 4.2. Plants Samples

In the experiments, *Sorghum bicolor* (L.) *Moench* (grain sorghum of the Eltonskoe variety), *Triticum aestivum* (wheat of the Moskovskaya 40 variety), and *Fragaria* L. (strawberry of the Ruyana variety) were used as test objects. In a number of experiments, seeds were treated with water activated by smoldering plasma. In a number of experiments, activated water was diluted with distilled water. In laboratory experiments, 100 seeds were used per group. The seeds were stratified at 4 °C and relatively high humidity for 2 weeks. The seeds were germinated in Petri dishes with filter paper at a constant temperature of 20 °C.

In the study of fusarium, we used seeds of *Sorghum bicolor* (L.) *Moench*, wheat *Triticum aestivum*, and strawberry *Fragaria* L. healthy (−) and affected by fusarium (+). The proportion of affected seeds was 98, 92, and 76% for wheat, sorghum, and strawberry, respectively. Fusarium blight was identified by the presence of the pathogens *Fusarium* sp. by microscopy and real-time PCR analysis. Samples were provided by All-Russian Phytopathology Research Institute, Russia.

### 4.3. DNA Extraction

To verify the microscopic data, we performed diagnostics of fusarium by real-time PCR. Isolation of genomic DNA from samples was performed using cetyl trimethylammonium bromide (CTAB method). A detailed description of the method is given in [44].

### 4.4. Real-Time PCR

Primers specific for these pathogens were used to identify Fusarium avenaceum, Fusarium graminearum, and Fusarium oxysporum in the respective samples (Table 6). All primers were synthesized at Evrogen (Moscow, Russia). The reaction mixture was prepared by mixing 5 µL of the ready-mixed qPCRmix-HS SYBR (Evrogen, Moscow, Russia) with a pair of target primers (1 µL each), 1 µL of the template DNA solution (1.28 × 10^2^ ng/mL), and Milli-Q water to a volume of 25 µL. The real-time PCR reaction was performed in an O-DTLITE 4S1 amplifier (DNA technology, Moscow, Russia). PCR for the identification of pathogens infestans was carried out according to the following protocol: denaturation 85 s at 94 °C, then 25 cycles 35 s at 95 °C, 30 s at 53 °C, and 30 s at 72 °C. Fluorescence intensity measurements were performed at the end of the 72 °C cycle. *C*t values, standard curves, and the corresponding correlation coefficients (R^2^) were automatically obtained using the Sequence Detection System v.1.2 software (Waltham, MA, USA) by interpolating *C*t values against the decimal logarithms of the initial DNA concentrations [45]. As a negative control, 2 µL of Milli-Q water was added to the reaction mixture instead of the DNA template.

### 4.5. Physicochemical Properties of Aqueous Solutions

Redox potential, pH, and electrical conductivity were measured on an S470 SevenExcellence high-precision measuring station (Mettler Toledo, Columbus, OH, USA). The recommended sensor electrodes InLab Expert Pro-ISM and InLab731-ISM (Mettler Toledo) were used. During measurements, aqueous solutions were mixed in a laminar mode using a magnetic stirrer (rotation frequency 3 Hz). All measurements were carried out at a solution temperature of 20 ± 1 °C. The experimental measurement details were described previously [48].

The concentration of molecular oxygen dissolved in water solutions was measured using an AKPM-1-02 polarograph (Bioanalytical systems and sensors, Moscow, Russia) [49]. The measurements took into account the atmospheric pressure, measured with a PRX-7001t (Casio, Tokyo, Japan), and the temperature of the samples, measured with a thermocompensating electrode. All measurements were carried out at a solution temperature of 20 ± 1 °C. The experimental measurement details were described previously [50].

The content of nitrate anions in the samples was determined using the Griss reagent according to the method described earlier [12]. To determine the concentrations of nitrate anions, the test solutions in a volume of 100 μL were applied to the wells of a 96-well flat-bottom polystyrene plate. Thereafter, a freshly prepared saturated solution of VCl_3_ (8 g/L in 1 M HCl) in a volume of 100 μL. Immediately thereafter, 100 μL of Griss reagent (1 M HCl containing 10 g/L of sulfanilamide and 1 g/L of N-1-naphthylethylenediamine hydrochloride) was added. The reaction was carried out in the dark at 37 °C for 1 h. After that, the optical density of the medium was measured at a wavelength of 546 nm using a Multiscan FC plate reader (TermoScintific, Vaanta, Finland). Sodium nitrate solutions of known concentration were used for calibration.

For the quantitative determination of hydrogen peroxide in aqueous solutions, a highly sensitive method of enhanced chemiluminescence in the luminol-p-iodophenol-horseradish peroxidase system was used [51]. The luminescence intensity was determined using a Biotox-7A chemiluminometer (ANO ICE, Moscow, Russia). For calibration, hydrogen peroxide solutions of known concentration were used. The initial concentration of hydrogen peroxide used for calibration was determined spectrophotometrically at a wavelength of 240 nm with a molar absorption coefficient of 43.6 (M^−1^ × cm^−1^). The “counting solution” contained: 1 cM Tris-HCl buffer pH 8.5, 50 µM p-iodophenol, 50 µM luminol, 10 nM horseradish peroxidase. The sensitivity of the method makes it possible to determine hydrogen peroxide at a concentration of less than 1 nM [52].

### 4.6. Field Experiment

The experiment was carried out on a sorghum crop of the “Eltonskoye” variety in a saline clay semi-desert of the Northern Caspian region, in the Pallasovsky district of the Volgograd region. The advanced farm of the region—SPK Plemzavod Krasny Oktyabr—was used as a production base. The sum of the average annual precipitation in this region corresponds to 280–300 mm. The soils were represented by a 3-membered solonetzic complex, including up to 50–60% of solonetzes. The average long-term productivity of virgin vegetation, according to research, was 12–15 c/ha. The yield of hay from the solonetz complex when mowing was 3–4 c/ha, with normalized grazing by livestock—5.6 c/ha.

The treatment of inoculum with activated water and the setting up of the experiment was carried out as follows. A solution for pre-sowing seed treatment was prepared by diluting activated water in a ratio of 1:500 with water from an open reservoir. The seeds were moistened by watering and mixing the herd of seeds and then keeping them covered for 14 h (for 1000 kg of seeds—5 L of solution with a working concentration). The sowing of the treated seeds was carried out without allowing them to dry out. Sowing area of the option using activated water—100 hectares; control—100 hectares. The sowing rate of sorghum seeds was 10 kg/ha.

### 4.7. Statistics

Data were analyzed using SigmaPlot 11 software (Palo Alto, CA, USA) and were presented as means ± SEM. Data from at least 3 independent experiments were used for averaging.

## 5. Conclusions

The results obtained allow us to draw the following conclusions:

1. Activated water can be attributed to the category of plant growth regulators of grain and fruit crops, simultaneously contributing to an increase in plant resistance to negative biotic and abiotic environmental factors.
2. High concentrations of activated water can partially or completely block the growth and development of plants. The positive effect of the drug is usually manifested when the stock solution is diluted 250–500 times.
3. Pre-sowing treatment of seeds with activated water affects the growth and development of sorghum during the entire growing season.
4. The effectiveness of the drug is manifested even when grown on poor saline soils and in drought conditions, in which the effect of fertilizers and most growth stimulants is weak due to lack of moisture.
5. Activated water in optimal concentration is an inexpensive, promising, environmentally friendly preparation for production. In extreme growing conditions, the device developed by us is of particular value and promising for use.

## Figures and Tables

**Table 1:** Changes in the physicochemical parameters of aqueous solutions after exposure to glow discharge plasma for 20 or 40 min.

Exposure Time	Measured Parameters					
	EC **, mS/cm	[O_2_], μM	pH	Redox, mV	NO_3_^−^, mM	H_2_O_2_, mM
0 min	7.3 ± 0.5	273 ± 5	6.7 ± 0.1	303 ± 7	<0.01	<0.01
20 min	16.4 ± 1.0 *	264 ± 7	7.8 ± 0.1 *	510 ± 32 *	10.98 ± 0.61 *	3.48 ± 0.43 *
40 min	24.9 ± 1.2 *	261 ± 8	8.3 ± 0.2 *	598 ± 26 *	22.05 ± 0.98 *	7.12 ± 0.68 *

* Statistical differences relative to control (*p* < 0.05); ** EC—electrical conductivity.

**Table 2:** Influence of aqueous solutions activated by glow discharge plasma on seed infestation with fusarium.

Microscopy, Seed Contamination Level, %						
	Sorghum		Wheat		Strawberry	
	+	−	+	−	+	−
Control	98	0	92	0	76	0
Deionized water	97	0	90	0	76	0
Activated solution	35	0	61	0	52	0
**RT-PCR, Seed Infection Rate, *C*t**						
Control	10	>40	17	>40	29	>40
Deionized water	16	>40	18	>40	28	>40
Activated solution	>40	>40	27	>40	35	>40

**Table 3:** Germination of sorghum seeds after short-term soaking in activated water.

	Soaking Time, h						
	1	3	5	7	9	11	24
Germination, %	80	85	93	91	81	69	0

**Table 4:** Main parameters of young sorghum sprouts 120 h after treatment.

	Germination, %	Root Length, mm	Sprout Weight, g
Control	80 ± 3	8.2 ± 1.2	6.1 ± 0.5
Activated solution	91 ± 2 *	9.7 ± 0.7	7.6 ± 0.2 *

* Statistical differences relative to control (*p* < 0.05).

**Table 5:** The main parameters of sorghum seedlings after 120 h from the moment of soaking in activated water and its solutions for 24 and 48 h.

Dilution Rate	Soaking Time, h	Germination, %	Root Length, mm
Control	24	78 ± 3	8 ± 2
	48	81 ± 3	13 ± 4
Without dilution	24	0	0
	48	0	0
1:250	24	54 ± 5 *	6 ± 2
	48	67 ± 4 *	14 ± 3
1:500	24	84 ± 2 *	12 ± 2 *
	48	87 ± 2 *	22 ± 5 *

* Statistical differences relative to control (*p* < 0.05).

**Table 6:** Primers used to *Fusariun* sp. identification.

Species and Target	Primers (F and R)	Ref
F. graminearu	5′-GTTGATGGGTAAAAGTGTG-3′	[46]
Intergenic Spacer of rDNA (IGS region)	5′-CTCTCATATACCCTCCG-3′	
F. avenaceum	5′-ATGGGTAAGGARGACAAGAC-3′	[46]
гeн translation elongation factor 1-alpha (TEF1)	5′-GGARGTACCAGTSATCATG-3′	
F. oxysporum	5′-CAGACTGGGGTGCTTAAAGTT-3′	[47]
specific fragment between the transcription factors Han and Skippy	5′-AACGCTAGGGTCGTAACAAA-3′	

## Data Availability

Not available.
